# Determination of Lamotrigine in Pharmaceutical Preparations by Adsorptive Stripping Voltammetry Using Screen Printed Electrodes

**DOI:** 10.3390/s8074201

**Published:** 2008-07-15

**Authors:** Olga Domínguez-Renedo, M. Encarnación Burgoa Calvo, M. Julia Arcos-Martínez

**Affiliations:** Departamento de Química, Área de Química Analítica, Facultad de Ciencias, Universidad de Burgos, Plaza Misael Bañuelos s/n, E-09001 Burgos, Spain; E-Mails: olgado@ubu.es; meburgoa@ubu.es

**Keywords:** lamotrigine, screen-printed electrodes, differential pulse adsorptive stripping voltammetry

## Abstract

This paper describes a procedure that has been optimized for the determination of lamotrigine by Differential Pulse Adsorptive Stripping Voltammetry (DPAdSV) using carbon screen-printed electrodes (CSPE) and mercury coated carbon screen-printed electrodes. Selection of the experimental parameters was made using experimental design methodology. The detection limit found was 5.0 × 10^-6^ M and 2.0 × 10^-6^ M for the non modified and Hg modified CSPE, respectively. In terms of reproducibility, the precision of the above mentioned methods was calculated in %RSD values at 9.83% for CSPE and 2.73% for Hg-CSPE. The Hg-coated CSPEs developed in this work were successfully applied in the determination of lamotrigine in pharmaceutical preparations.

## 1. Introduction

**Figure 1.**
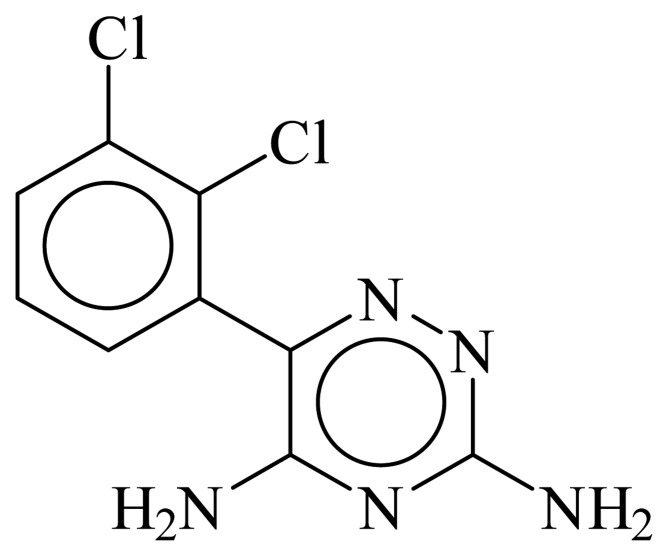
Chemical structure of lamotrigine.

Lamotrigine (LTG), 3,5-diamino-6-(2,3-dichlorophenyl)-1,2,4-triazine (Figure 1) is a new-generation antiepileptic drug registered for treatment of patients with refractory partial seizures with or without secondary generalization [1, 2]. It acts by inhibiting presynaptic voltage-sensitive sodium channels and excitatory neurotransmitter release.

HPLC [3-7] and capillary electrophoresis [8] are among the different techniques generally used for the measurement of LTG concentrations in pharmaceutical products and biological fluids. Despite the presence of redox groups in this molecule, only one article in currently available in the literature describes the analysis of LTG by means of electrochemical techniques using a HMDE electrode and adsorptive stripping voltammetry [9]. Electroanalytical techniques and screen-printing (thick-film) technology has made it possible to mass-produce inexpensive disposable electrodes for use with electrochemical instruments [10-14]. Their use in potentiometric, amperometric and voltammetric devices have been reported for the detection of heavy metals such as copper [15-18], lead [19-22], cadmium [20, 22] and mercury [17] although there are few references in bibliography of their use in the determination of drugs [23].

Numerous experimental variables can affect the response when using stripping voltammetry techniques, calling for a process of optimizing the variables which will enable accurate measurements under the best possible conditions. In the improvement of any analytical procedure, special precautions need to be taken when choosing the experimental conditions, especially when it is a matter of trace level determination of species. An appropriately designed experiment [24, 25] provides signals of far superior quality to those measured in an experiment that has not been optimized. Likewise, the use of experimental designs allows exploring a wide experimental range for a reduced number of experiments. They are more efficient than the “one-at-a-time” optimization of experimental variables in analytical techniques [26-30]. As a result, in our work, experimental design has been used to establish appropriate experiments that will lead to the optimization of the influencing variables, such as, potential, deposition time (E_dep_, t_dep_) and pH value.

## 2. Results and Discussion

### 2.1. Stripping voltammetry of lamotrigine at the non modified CSPE

**Figure 2.**
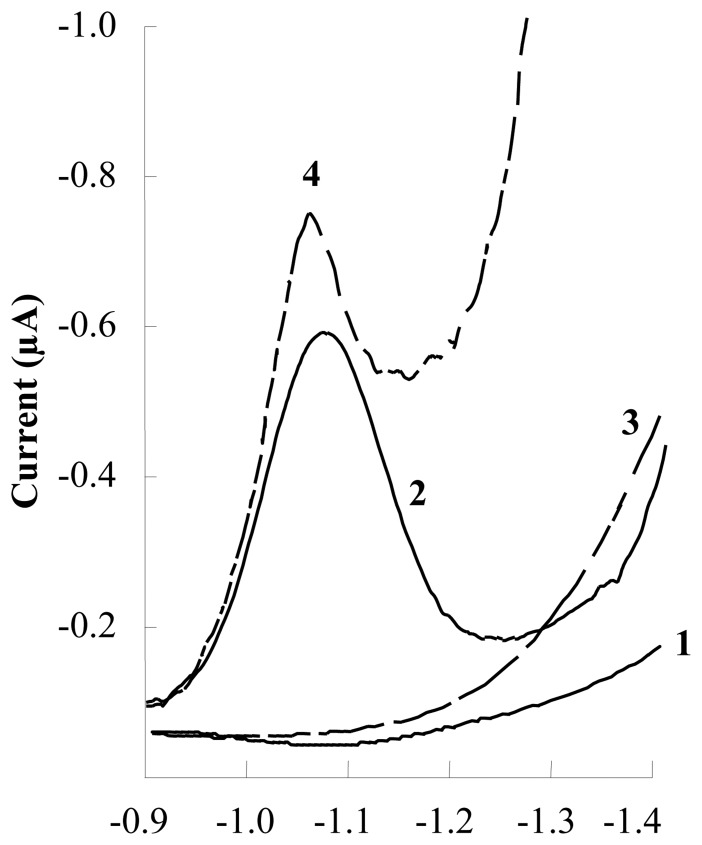
Differential pulse voltammograms obtained in Britton-Robinson (pH 5). Hg-CSPE: t_dep_ = 105s, E_dep_ = 0.40V (1) Blank (2) [LTG] = 1.5 × 10^-5^ M. ……… CSPE: t_dep_ = 28s, E_dep_ = 0.05V, (3) Blank (4) [LTG] = 1.5 × 10^-5^ M.

When differential pulse adsorptive stripping voltammetry (DPAdSV) analysis of LTG is carried out, using carbon screen-printed electrodes (CSPE) as working electrodes, a well defined reduction peak at − 1.16 V is observed (Figure 2).

This peak is due to the following reduction process [9]: 
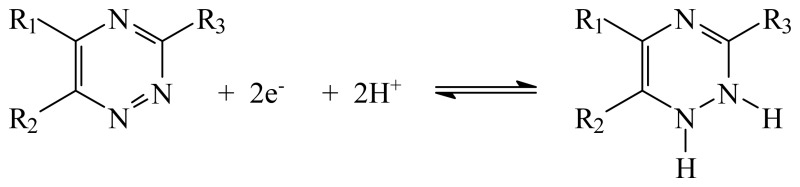


Judging from the response obtained, peak intensity (i_p_), was notably influenced by variables such as deposition time, t_dep_, deposition potential, E_dep_, and pH, so an experimental design was used to optimize these parameters.

Prior to the experimental design stage, previous assays had shown that a very low electrochemical signal was obtained for pH values very different from 5.5. It was, therefore, decided to fix this factor and perform the optimization of the two remaining factors. A central composite design was chosen for this stage, its purpose being to arrange the factors and their interactions according to their influence on the peak current. These factors were E_dep_ and t_dep_. Subsequently, experiments with all possible combinations were carried out. The values which correspond to the high (+) and low (-) levels and to the central point (0) for each factor were as follows,

$$\begin{array}{lll} {\text{E}}_{\text{dep}}(+)=+0.30\;\text{V} & \;{\text{E}}_{\text{dep}}(-)=-0.30\;\text{V} & \;{\text{E}}_{\text{dep}}(0)=0\;\text{V}\\ {\text{t}}_{\text{dep}}(+)=50\text{s} & \;{\text{t}}_{\text{dep}}(-)=10\;\text{s} & \;{\text{t}}_{\text{dep}}(0)=30\;\text{s} \end{array}$$

**Figure 3.**
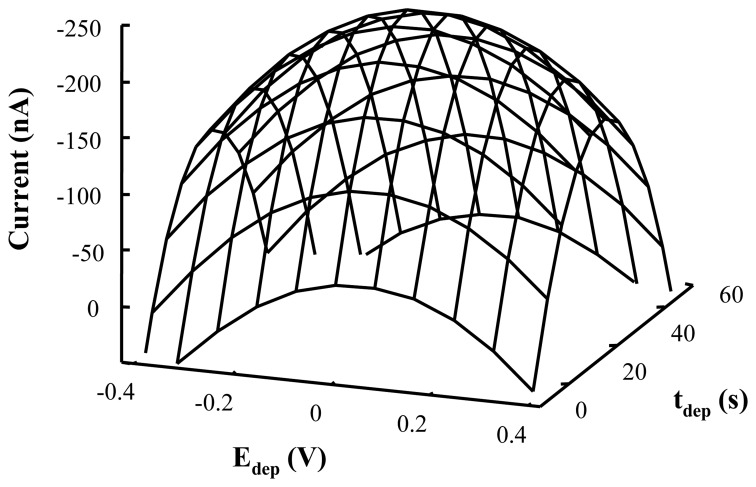
Response surface for the 2^2^ central composite design for optimization of experimental variables in LTG determination by DPAdSV using CSPE electrodes.

The response to be optimized was the intensity (-i_P_), of a lamotrigine sample at a concentration of 2.0 × 10^-5^ M at a potential peak of − 1.06 V. From the analysis of the variance (ANOVA) of the data (Table 1), it can be seen that a second order function is adequate to model the data because the lack of fit is not significant at the 95 % confidence level. It can also be deduced that the only significant factors are the AA and the BB interaction. However, a maximum can be observed in the response surface obtained for this design (Figure 3).

As a result of the above discussion, the optimum conditions for the determination of LTG by means of DPAdSV using the non modified-CSPE are:

$$\begin{array}{ccc} \text{pH}=5.50 & {\text{t}}_{\text{dep}}=28\;\text{s} & {\text{E}}_{\text{dep}}=0.05\;\text{V} \end{array}$$

### 2.2. Stripping voltammetry of lamotrigine in Hg- coated CSPE

In this case, in order to carry out the analysis of LTG, the electrode was modified by depositing a mercury film on its surface.

When the analysis of LTG is performed by means of DPAdSV using Hg coated CSPE a reduction peak at - 1.06 V is observed (Figure 2). In this case, an optimization of the influencing experimental variables was also carry out.

Previous assays showed that the intensity of the electrochemical response for LTG increased when positive E_dep_ were used, however the background noise suffered an important increasing when the potential of deposition was changed towards very positive values . For this reason, the deposition potential was fixed at 0.40 V due to the suitable quality of the signal obtained for this value.

The two remaining factors (pH and t_dep_) were optimized by means of a 2^2^ central composite design. The response to be optimized was the peak intensity obtained for a 1.5 × 10^-5^ M LTG solution. In the ANOVA test shown in Table 2 it can be seen that AA and BB interactions are both significant factors at a 95% confidence level, therefore, pH and t_dep_ have to be considered as influence variables.

**Figure 4.**
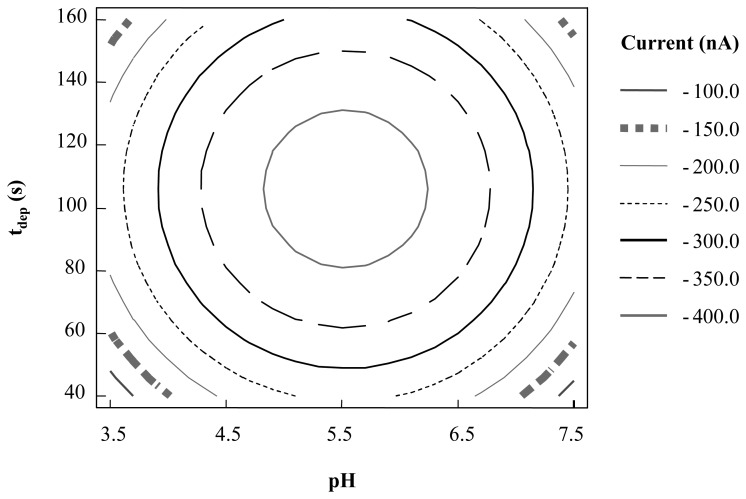
Level curves for the 2^2^ central composite design for optimization of experimental variables in LTG determination by DPAdSV with Hg film modified CSPE.

However, a maximum for the electrochemical response can be observed in Figure 4 which corresponds to the following values for the variables to be optimized:

$$\begin{array}{cc} \text{pH}=5.53 & \;{\text{t}}_{\text{dep}}=105\;\text{s} \end{array}$$

The electrochemical signal obtained with the Hg-coated CSPE is higher than the one obtained with the non modified CSPE. This fact allows the analysis of LTG at pH values different from 5.5 when using Hg modified CSPE. Nevertheless, it can be seen that even altering the pH the optimal value obtained is close to 5.53.

### 2.3. Calibration and Detection Limit

#### 2.3.1. Non modified CSPE

In order to determine the limit of detection of both procedures a calibration was performed, at low levels of concentration, using least-median-squares regression (LMS) to detect the existence of anomalous points [31], which might have led to incorrect adjustments altering the sensitivity and the detection limit. The criterion is to minimize the median of squares of the differences between the experimental and the calculated values. LMS Regression has the advantage of being able to detect anomalous points regardless of whether they are outliers or leverage points, seeking a linear range in which at least 50% of the data are aligned.

The strategy followed consisted of two steps. In the first, the LMS regression was used to detect anomalous points, taking outliers to be points where the absolute value of the standardized residual was greater than 2.50 and leverage points as those where the absolute value of their resistant diagnostic was greater than 2.50. When both of these parameters were above 2.50, the point was considered as an outlier-leverage. In a second step, the anomalous points detected in this way were eliminated and a regression based on the ordinary least squares (OLS) criterion was carried out, to obtain optimal precision and accuracy of both slope and intercept.

The calibration equation obtained by DPASV for standard solutions containing LTG concentrations of between 5.0 × 10^-6^ and 2.1 × 10^-5^ M was:

$$\begin{array}{c} -\text{IP}=-421.8+5.23\times 10^{7}{\text{C}}_{\text{LTG}}\\ \left({\text{R}}^{2}=0.99\;\text{and Standrad Deviation}\;({\text{S}}_{\text{yx}})=19.68\right) \end{array}$$

A key feature of an analytical method is the detection limit, the smallest concentration of the analyte that can be detected to a specified degree of certainty. The calculation of the detection limit, based on the variability of ten samples with a very low analyte concentration, was calculated according to [32] and ISO 11843-2 [33]. At the chosen probability level of 95% (α = β = 0.05), the detection limit was 5.0 × 10^-6^ M.

#### 2.3.2. Hg-coated CSPE

The calibration equation obtained by DPASV for standard solutions containing LTG concentrations of between 2.0 × 10^-6^ and 5.0 × 10^-6^ M was:

$$\begin{array}{c} -\text{IP}=-100.93+56.36\times 10^{6}{\text{C}}_{\text{LTG}}\\ \left({\text{R}}^{2}=0.99\;\text{and Standrad Deviation}\;({\text{S}}_{\text{yx}})=0.08\right) \end{array}$$

In this case, at the chosen probability level of 95% (α = β = 0.05), the detection limit was 2.0 × 10^-6^ M.

### 2.4. Precision

This parameter was calculated in terms of reproducibility. A series of 5 measurements of samples containing 1.5 × 10^-5^ M of LTG were carried out obtaining a % RSD value of 9.83 % for CSPE and 2.73 % when a Hg-coated CSPE was used. So, the Hg film modified CSPE resulted to be more precise.

From the above described results it can be deduced that the Hg-coated-CSPE is more useful for the analysis of LTG in terms of precision. For this reason this electrode has been chosen for the analysis of LTG in real samples.

### 2.5. Linear Range

The Hg-coated CSPE constructed as has been described in the previous sections resulted to offer a linear response in the range comprised between 2.0 × 10^-6^ and 1.8 × 10^-5^ M (-I_p_ = 141.59 + 40.60 × 10^6^ C_LTG_; R^2^ = 0.99 and Standard Deviation (S_yx_) = 14.43).

### 2.6. Determination of lamotrigine in real samples

The concentration of lamotrigine in commercial capsules of LAMICTAL^®^ (GlaxoSmithKline) with a known concentration of analyte, was determined by standard addition using the DPAdSV using the mercury film modified CSPE, so as to evaluate the accuracy of the proposed method.

Good agreement was obtained between the amount found by the developed method (24.5 ± 0.8) mg (n=3, α = 0.05) and the value supplied by the manufacturer (25 ± 1.2 mg). These results were also checked using the HPLC method described in [4] as a reference technique obtaining (25.2 ± 1.1) mg (n=3, α = 0.05).

## 3. Experimental Section

### 3.1. Reagents and chemicals

Analytical grade chemicals not subjected to any further purification processes were used. All solutions were prepared with deionized water obtained using a Barnstead NANO Pure II system. Nitrogen (99.99%) was used to remove dissolved oxygen.

Electrodag PF-407 A (carbon ink), Electrodag 418 SS (silver ink), Electrodag 6037 SS (silver/silverchloride ink) and Electrodag 452 SS (dielectric ink) were supplied by Achenson Colloiden (Scheemda, The Netherlands).

Solutions of lamotrigine were prepared by dissolving appropriate amounts of lamotrigine (Sigma, Steinheim, Germany) in water.

Britton-Robinson solutions were used as buffers. A 0.04 M Britton-Robinson buffer solution for the o-boric, o-phosphoric and acetic acids was prepared using Merck analytical grade reagents. Solutions of different pH values were prepared from this by the addition of 0.2 M sodium hydroxide (analytical-reagent grade, Merck, Darmstadt, Germany).

Commercial capsules of LAMICTAL^®^ were obtained from GlaxoSmithKline. The excipients of the tablet are: blackcurrant flavour, calcium carbonate, low-substituted hydroxypropylcellulose, magnesium aluminium silicate, magnesium stearate, povidone, saccharin sodium and starch glycolate).

In order to determine the concentration of LTG in LAMICTAL^®^ tablets the following procedure was carried out: Ten tablets were pulverized with a pestle and a portion of the resulting powder was dissolved in water. The insoluble portion of the tablet was eliminated by filtration.

### 3.2. Apparatus

Screen-printed electrodes were produced on a DEK 248 printing machine (DEK, Weymouth, UK) using polyester screens with appropriate stencil designs mounted at 45° to the printer stroke.

Voltammetric measurements were taken using a μAutolab (Eco Chemie). The following values for the instrumental parameters were used: pulse amplitude, -62 mV, staircase size, 4 mV and duration of the pulse in the staircase potential sweep, 500 ms.

The pH of the solution was measured with a Crison Model 2002 (Barcelona, Spain) pH meter.

### 3.3. Software

Data analysis was processed with a STATGRAPHICS PLUS software package [34] for the experimental design process and PROGRESS [31] for the robust regression.

### 3.4. Construction of Screen-printed Electrodes

**Figure 5.**
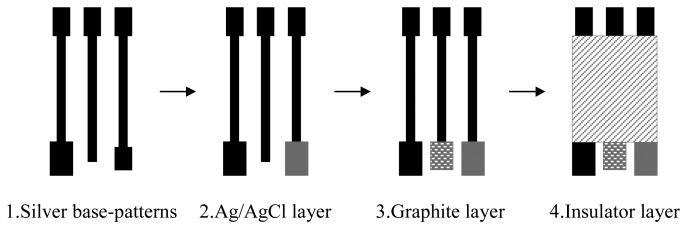
Schematic diagram of the sensor preparation procedure.

In this study, hand-made screen-printed electrodes were used in the determination of LTG. A three-electrode configuration (working, reference and an auxiliary electrodes) was constructed for the determination of LTG. Since shape, surface area and spatial arrangement of the electrodes significantly influence the quality of the analytical response, the electrode system design deserves special attention. The design employed in this work is shown in Figure 5. In order to assemble the screen-printed electrodes, successive layers of different inks were printed onto a PVC strip substrate (30 mm × 15 mm, 0.5 mm thick) using four different screens with an appropriate stencil in order to reach the required design (Figure 5). The printing procedure was as follows:

1) Firstly, three parallel conducting base-patterns were printed with the commercial silver ink to give the screen-printed electrodes an effective conductive nature and were then cured for 15 minutes at 90 °C. The base-pattern at the left was used as the counter electrode.
2) A silver/silver chloride reference electrode was printed using silver/silver chloride ink on the silver base-pattern at the right, as can be seen in Figure 5, and then cured for 15 minutes at 90 °C.
3) The working electrode was formed by printing a graphite layer over the silver base-pattern at the center using commercial graphite ink and was then cured for 15 minutes at 90 °C.
4) Finally, excepting the surface of the three electrodes and the electrical connection at the reverse end of the sensor strip, an insulator layer was printed over the sensor strip and then cured by UV radiation.

### 3.5. Mercury film preparation

In a separate process, the mercury film was coated over the screen-printed working electrode surface, using a solution containing 800 mg L^-1^ of HgCl_2_. Good analytical signals in the analysis of LTG were obtained when the deposition of the mercury film was performed by applying a potential of - 0.9 V during 600 s under stirring in 1.3 M HCl solution.

### 3.6. Stripping voltammetry measurements

Voltammetric measurements were taken using the following procedure: the solution was purged using nitrogen, and stirred for 300 s, the deposition potential was then applied for the time and potential as determined for each experiment. The solution was left to rest for an equilibrium time of 5 s, then a cathodic scan from 0 V (initial potential) to − 1.40 V (final potential) was started and the voltammogram recorded, using a potential step of 6.00 mV. The modulation time was 0.04 s and the interval time of the applied pulses was 0.60 s.

Non modified CSPEs electrodes can be used only for one measurement whereas Hg-coated SCPE can be used several for several measurements.

## 4. Conclusions

The Hg-modified carbon screen printed electrodes developed in this work present an interesting method for the analysis of LTG. One of the interesting contributions of this work is the viability of performing analysis of LTG in pharmaceutical preparations in an easy way using simple regression because no effect from the sample matrix was found. This analysis is more difficult when using other electrodes such as a HDME where a multivariate calibration was necessary [9]. Moreover, the proposed method is a more environmental friendly form to determine LTG.

## Figures and Tables

**Table 1.** ANOVA of the data obtained with the 2^2^ central composite design for optimization of experimental variables in LTG determination with CSPE by DPAdSV. [LTG] = 2.0 × 10^-5^ M, pH = 5.5

Effect	SS*	DF*	MS*	F_ratio_*	P_level_*
A: E_dep_	2478.870	1	2478.870	10.900	0.081
B: t_dep_	1407.510	1	1407.510	6.190	0.131
AA	18565.500	1	18565.500	81.670	0.012(a)
AB	5.664	1	5.664	0.020	0.889
BB	50502.10	1	50502.100	222.160	0.004(a)
Lack-of-fit	4720.510	3	1573.500	6.920	0.129
Pure error	454.648	2	227.324		
Total	64957.800 R^2^ = 92.033	10			

* SS, sum of squares; DF, degrees of freedom; MS, mean squares; F_ratio_: MS_factor_/MS_error_; P_level_, probability level.

(a) Significant factor at α = 0.05.

**Table 2.** ANOVA of the data obtained with the 2^2^ central composite design for optimization of experimental variables in LTG determination with Hg film modified CSPE by DPAdSV. [LTG] = 1.5 × 10^-5^ M, E_dep_ = 0.40 V

Effect	SS*	DF*	MS*	F_ratio_*	P_level_*
A: E_dep_	292.47	1	292.47	0.10	0.78
B: t_dep_	5943.26	1	5943.26	1.99	0.29
AA	201566.00	1	201566.00	67.65	0.01(a)
AB	1.55	1	1.55	0	0.98
BB	104949.00	1	104949.00	35.23	0.03(a)
Lack-of-fit	8847.37	3	2949.12	0.99	0.54
Pure error	5958.75	2	2979.38		
Total	262926.00 R^2^ = 94.37	10			

* SS, sum of squares; DF, degrees of freedom; MS, mean squares; F_ratio_: MS_factor_/MS_error_; P_level_, probability level.

(a) Significant factor at α = 0.05.
